# Herpetic optic perineuritis presenting as classic abducens nerve palsy

**DOI:** 10.1002/ccr3.2571

**Published:** 2019-12-03

**Authors:** Muhammad Abbas Abid, Mustafa Mughal, Peter Capelli, Muhammad Bilal Abid

**Affiliations:** ^1^ Department of Otolaryngology - Head & Neck Surgery Johns Hopkins University School of Medicine Baltimore MD USA; ^2^ Department of Internal Medicine Liaquat College of Medicine & Dentistry Karachi Pakistan; ^3^ Medical College of Wisconsin Milwaukee WI USA; ^4^ Divisions of Hematology/Oncology & Infectious Diseases Department of Medicine Medical College of Wisconsin Milwaukee WI USA

**Keywords:** abducens nerve palsy, diplopia, herpes zoster ophthalmicus, herpes zoster optic perineuritis

## Abstract

Optic perineuritis is a rare manifestation of herpes zoster ophthalmicus (HZO). Relative afferent pupillary defect (RAPD) is an important early clue to an impending nerve involvement, and robust clinical examination allows early detection of such rare metachronous manifestation of cutaneous HZ and institution of timely management for such sight‐threatening conditions.

## Graphical abstract

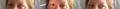

A 55‐year‐old woman presented acutely with monocular pain, blurring, and double vision in the right eye. Physical examination was remarkable for deviation of the right eye towards midline, and she was unable to abduct it laterally (Figure 1). Other visual function tests, including visual acuity (20/20) and visual fields, were normal. Although there was no exophthalmos or optic disk swelling on fundoscopy, right eye exhibited a relative afferent pupillary defect (RAPD). Color vision, on the Ishihara chart, was normal in both eyes. She was found to have an isolated abducens nerve palsy clinically and enhancement of right optic nerve sheath, without optic nerve damage, radiographically (Figure 2). Subclinical optic nerve damage might have been demonstrated by more sensitive color vision tests such as FM 100‐hue.

**Figure 1:**
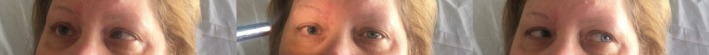
Examination of extraocular movements across cardinal directions shows an inability to abduct the right eye. The figure also shows scabbed rashes over the ophthalmic branch of the trigeminal nerve (V1 distribution)

**Figure 2:**
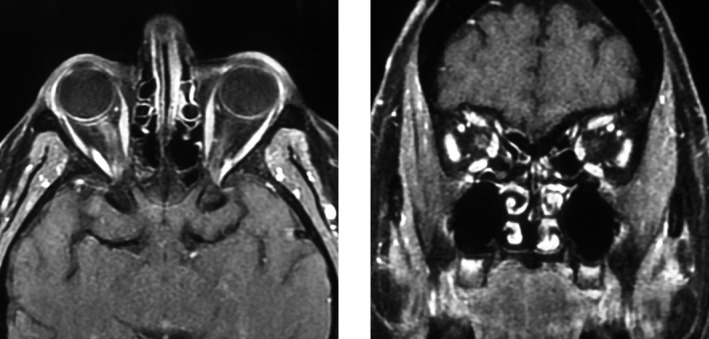
MRI orbits, axial and coronal sections, show enhancement of right optic nerve sheath

The neuro‐ophthalmological symptoms were preceded by vesicular eruption over her eye/forehead (Figure 1) and ipsilateral frontal headache. She was treated with IV acyclovir for 7 days followed by another week of oral valacyclovir. Postdischarge, patient's rash and headache resolved while diplopia persisted. Four months after the infection, the patient's diplopia had progressively improved. Optic perineuritis, a rare, sight‐threatening complication of herpes zoster ophthalmicus, can occur simultaneously with an acute vesicular skin eruption.^1^, ^2^ RAPD may be an early clinical clue, allowing prompt treatment, and could prevent the progression of visual symptoms.

## CONFLICT OF INTEREST

None declared.

## AUTHORS’ CONTRIBUTIONS

MAA: wrote the manuscript; MM and PC: cowrote the manuscript; MBA: provided clinical care and supervised the study. All authors performed a critical revision of the manuscript and approved the final version of the manuscript.

## INFORMED CONSENT

Informed consent was obtained from patient prior to publication.
